# What do primary care staff think about patients accessing electronic health records? A focus group study

**DOI:** 10.1186/s12913-022-07954-y

**Published:** 2022-04-29

**Authors:** Gemma Louch, Abigail Albutt, Kate Smyth, Jane K. O’Hara

**Affiliations:** 1 Bradford Institute for Health Research, Bradford Teaching Hospitals NHS Foundation Trust, Bradford, BD9 6RJ UK; 2 NIHR Yorkshire and Humber Patient Safety Translational Research Centre, Bradford, BD9 6RJ UK; 3 Lancashire Teaching Hospitals NHS Foundation Trust, Preston, UK; 4 School of Healthcare, University of Leeds, Leeds, LS2 9DA UK

**Keywords:** Primary care, Electronic health records, Patient involvement, Patient safety

## Abstract

**Background:**

Patients have expressed a growing interest in having easy access to their personal health information, and internationally there has been increasing policy focus on patient and care records being more accessible. Limited research from the UK has qualitatively explored this topic from the primary care staff perspective. This study aimed to understand what primary care staff think about patients accessing electronic health records, highlighting errors in electronic health records, and providing feedback via online patient portals.

**Methods:**

A focus group study involving 19 clinical and non-clinical primary care staff.

Primary care practices were purposively sampled based on practice size and the percentage of patients using online services. Data were analysed inductively using reflexive thematic analysis.

**Results:**

Three themes were generated: (1) Information – what, why and when? (2) Changing behaviours and protecting relationships, and (3) Secure access and safeguarding. The emotional considerations and consequences for staff and patients featured prominently in the data as an overarching theme.

**Conclusions:**

Primary care staff described being invested and supportive of patients accessing their electronic health records, and acknowledged the numerous potential benefits for safety. Uncertainty around the parameters of access, the information available and what this might look like in the future, processes for patients highlighting errors in records, relational issues, security and safeguarding and equitable access, were key areas warranting examination in future research.

## Background

Research suggests that the digitalisation of health records can have a positive impact on patient satisfaction, preventative self-care, and enhanced patient safety through identifying medication errors for example [1, 2]. However, potential risks and unintended consequences, often resulting from challenges around design, usability, implementation and health professional use are acknowledged [3]. A recent systematic review and meta-analysis evaluated the impact of sharing electronic health records (EHRs) with patients and mapped the findings across six domains of quality of care [4]. The review highlighted that patient access to EHRs can enhance effectiveness and patient safety, but that further research is needed to be more confident in these findings.

Patients have expressed a growing interest in having easy access to their personal health information [5, 6], and internationally there has been increasing policy focus on patient and care records being more accessible [7–9]. In Sweden for example, as part of the national e-health strategy, by 2020 residents from 16 years of age were projected to have access to all information documented in county funded health and dental care [10, 11]. In Canada, health care institutions have made progress on patient access to records [12, 13], however, there is still great variation in the type and timing of information released [14].

From a UK policy perspective, this topic has been gaining momentum [7]. Since 2014, patients have already been able to access their electronic summary record, but a 2016 study reported that only 0.4% of patients are accessing this, and only 0.1% of patients have access to their full GP record online [15]. The NHS App enables patients to book and manage appointments at their GP practice, order their repeat prescriptions and view their GP medical record securely. The NHS App pilot testing phase took place in 2018 and included 3000 patients from 34 GP practices, with most users indicating they used the app to view their medical record [16].

Initiatives supporting open patient access to EHRs have been piloted successfully in many countries, including the USA, Sweden and the UK [17–19]. One such example is OpenNotes, a national movement that invites patients to read their clinicians’ notes online, predominantly in use in the USA. The findings of an OpenNotes demonstration and evaluation project revealed that most patients chose to read their notes and reported benefits from doing so, and crucially, doctors reported few workload effects [20]. Following this 12-month project, 99% of patients indicated that they wanted to continue to have access to their notes online and all of the doctors decided to continue with the initiative. The uptake and implementation of OpenNotes has since grown, and more recent research using pre- and post-implementation surveys at three USA sites concluded that “…despite concerns about errors, offending language or defensive practice, transparent notes overall did not harm the patient–doctor relationship. Rather, doctors and patients perceived relational benefits” [21]. Furthermore, a survey of 136,815 patients with OpenNotes found that patients who read ambulatory notes online recognised mistakes, and a large amount of these were perceived to be serious, with older and sicker patients being twice as likely to highlight a serious error compared with younger and healthier patients [22]. Recent qualitative research examining the factors influencing the development and implementation of patients’ access to EHRs [23] identified resistance from health care professionals as a key barrier, including concerns around patient misunderstanding, increased workload and implications relating to professional communication. This echoed concerns highlighted in previous research also from the health care professional perspective [24]. Indeed, research by Blease and colleagues’ which considered whether patient access to clinical notes changes documentation, suggested that although patients’ and clinicians’ experiences of the practice are generally positive, there are some potential risks [25]. For example, clinicians have reported changes to documentation practices such as less detailed notes, and removal of language that could be perceived as critical. The authors described how medical records may be used as form of ‘cognitive scaffolding’ to support memory and diagnostic reasoning, and that approaches to documentation by clinicians to minimise or prevent anxiety for patients have the potential to challenge the essential nature of record keeping.

Digital technology in the NHS has expanded significantly as a result of the COVID-19 pandemic, with increasing numbers of patients using remote health services [26]. Recently, NHS Digital announced plans to accelerate patient access to their records through the NHS App and other online services from December 2021,^1^ including plans for patients to be able to read all new entries in their health record and plans for patients to be able to request historic coded records. Relatedly, there is promising evidence that patients’ access to their notes may have a positive impact on challenges that developed, or were exacerbated by the COVID-19 pandemic, for example by addressing health disparities, strengthening clinician-patient exchanges, and supporting patient and family involvement [27].

Concurrent with this focussed attention on enabling patient access to EHRs and online patient portals, over the last decade a growing body of research has emerged around patient involvement in healthcare and more specifically, patient safety [28]. In recognition of this, it is now widely accepted that patients can willingly and meaningfully be involved in the safety of their care [28, 29]. Recent efforts have begun to concentrate on more novel ways of involving patients. For instance, in a hospital setting, patient feedback about safety has been collected using validated reporting tools on handheld devices, facilitated by hospital volunteers [30], and online reporting repositories such as Care Opinion (https://www.careopinion.org.uk/) are being used more frequently. We feel there is value in bringing these bodies of literature together to explore how we might advance patient involvement in patient safety in a primary care setting in the context of EHR access.

Online patient portals which include EHRs are acknowledged as a promising mechanism to support greater patient engagement. Indeed, a 2015 ‘state of the science review’ concluded that further work is needed to understand how health care leaders, policy makers, and designers can encourage adoption of patient portals. The review emphasised that to encourage adoption of patient portals, features of these systems need to align with patients’ and providers’ information needs and functionality [31].

In this study we aimed to explore patient access to EHRs and digital approaches to gathering quality and safety information from patients about their care, from the perspective of primary care staff in the UK.

### Research questions

Research question 1: What is the primary care staff perspective on patients accessing and viewing their electronic health record?

Research question 2: What is the primary care staff perspective on patients highlighting errors in their electronic health record and providing feedback via online patient portals?

## Method

This paper was drafted in line with the consolidated criteria for reporting qualitative research (COREQ) [32] where appropriate.

### Patient involvement in the design and conduct of the study

We sought advice from the Yorkshire Quality and Safety Research (YQSR) Group patient panel on the design and focus of the study, and one of our Lay Leaders from the NIHR Yorkshire and Humber Patient Safety Translational Research Centre (KS) provided input throughout the study, and has contributed to drafting this paper.

### Design

A pragmatic qualitative approach was adopted involving focus groups and interviews with clinical and non-clinical primary care staff. A focus group method was chosen as we were interested in group interaction [33]. The potential impact of increased patient access to online services may have implications for both clinical and non-clinical staff, for instance, non-clinical staff may have responsibilities to respond to patient queries regarding inaccuracies. By inviting and including both non-clinical and clinical staff in the focus groups together, we intended discussions to prompt participants to consider their ideas further to produce insights that may not have arisen without the mix of staff groups and interaction. The topic guide was informed by a previous study that explored patients’ views on reporting errors in their EHR and providing feedback via online practice portals [34]. The topic guide followed a semi-structured format with broad questions around patients viewing their EHR, patients reporting errors in their EHR, and patients providing feedback via online practice portals. The questions and discussion explored current practice as well as potential challenges and opportunities.

### Setting and sample

Nineteen participants were recruited from six primary care practices in Yorkshire (UK) between October and November 2019. We purposively sampled practices to reflect status in terms of Patient Online Management Information (POMI)^2^ (i.e. percentage of patients using online services) and practice size.^3^

We conducted four focus groups, one dyadic interview and one single interview. The sample included a range of clinical and non-clinical primary care staff (see Table 1) and was predominantly female (94.7%). The average age of the sample was 41.37 years (Standard Deviation = 11.69; range = 38), average years in clinical practice = 17.09 (Standard Deviation = 12.14; range = 36), and average number of months in current role = 84.53 (Standard Deviation = 87.34, range = 287). In terms of ethnicity, 68.4% of the sample identified as White British, 21.2% as Asian/Asian British Pakistani, 5.3% as Asian/Asian British Indian and 5.3% as White and Asian.

**Table 1:** Participant job titles and duration of focus groups and interviews

	Participant job titles	Duration (minutes)
Focus group 1	GP Partner, FY2, Healthcare Assistant and Receptionist, Practice Manager	41:08
Focus group 2	Patient Service Manager, GP, Medical Secretary, Lead Practice Nurse	28:51
Focus group 3	GP Partner, GP, Healthcare Assistant, Advanced Nurse Practitioner	37.11
Focus group 4	GP Principal, Patient Service Manager, Practice Nurse, Patient Service Assistant	28:10
Dyadic interview	Administrator, Practice Manager	29:45
Single interview	Office Manager	16:06

*FY2* Foundation Doctor Year 2

### Procedure

GP practices were invited to participate in the focus group study via email. If a practice was interested in participating, the study information sheet was provided so this could be distributed to potential participants, and a date and time agreed. Both clinical and non-clinical staff were invited to participate. We did not limit the number of staff attending focus groups, the size of the focus groups reflects the availability of staff who wished to participate. At two practices, it was not feasible to conduct a focus group so dyadic and one-one interviews were utilised. Written informed consent was obtained from participants as well as brief demographic information, and the focus groups and interviews were audio recorded. Focus groups and interviews took place in a private room at participating practices, facilitated by AA, a female, PhD, academic researcher with a background in psychology and health services research. AA had no prior relationship with those who participated in the focus groups and interviews. GP practices received remuneration to cover staff time and participants were offered a certificate confirming participation in the study, which could be used as evidence in revalidation or appraisal portfolios.

### Ethics and governance

Ethical approval was sought from the University of Leeds, School of Medicine Research Ethics Committee (SoMREC), reference MREC 18-076, and HRA (Health Research Authority) approval was obtained, reference 19/HRA/3966.

### Data analysis

The focus groups and interviews were digitally recorded and transcribed verbatim. Data were analysed inductively using reflexive thematic analysis [35, 36] through a process of data familiarisation, generating codes, initial theme development, reviewing themes, defining and naming themes. We used NVivo 10 software to facilitate data management and analysis. Two researchers (GL – Psychologist and Health Services researcher and AA – Psychologist and Health Services researcher) familiarised themselves with the data by listening to the audio recordings and reading and re-reading the transcripts to develop provisional codes collaboratively. GL applied the framework to all transcripts and met regularly with AA throughout the process of coding and theme development.

## Findings

**Fig. 1:**
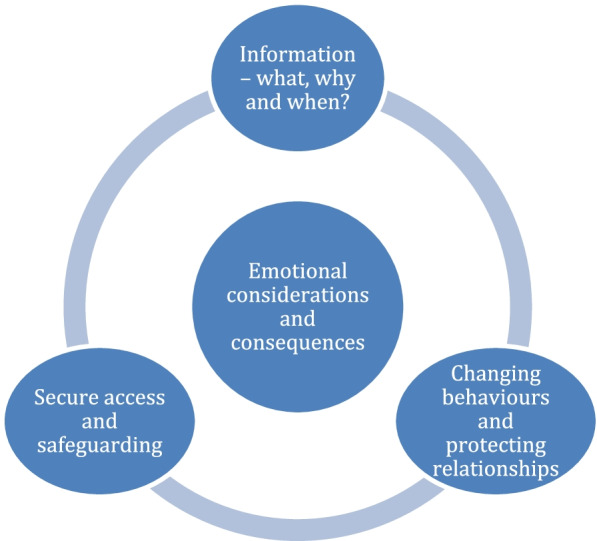
Overview of themes

Three themes were generated: (1) Information – what, why and when? (2) Changing behaviours and protecting relationships, and (3) Secure access and safeguarding (see Fig. 1). The emotional considerations and consequences for staff and patients featured prominently in the data and we therefore consider this to be an overarching theme, reflected within all four themes. We present a descriptive account of the themes with supporting excerpts below.

### Theme: information – what, why and when?

Staff recognised the potential benefits for patients and the practice resulting from increased patient access to EHRs, however, there was a great deal of discussion about what information could be viewed in the future if current access was expanded and if uptake became more widespread. These discussions were predominantly focussed around three areas: the level of information, the use of information and historical information.

#### Level of information

Regarding the level of information, staff expressed a greater degree of comfort with the prospect of patients viewing coded as opposed to free text information:

*‘Um, it’s a good thing, so that they know exactly what’s happening. I think they should be able to see most of the stuff but not the, the free text information because, quite often that can be doctor’s thought process down on there…’ *GP Partner (Focus group 1)

Nonetheless, it was acknowledged that at present, even certain coded information might not be in an accessible format, and this could result in misinterpretation:

*‘I think as well some of our coding is a bit out of date, so I find when I am going through like my post-natal checks, that I am tidying up if you like the major coding and…where I am just kind of like “oh that doesn’t need to be there” and I think there might….I think our notes aren’t necessarily tidy enough in some respects just because of the way things have been coded in the past..’ *GP (Focus group 3)

#### The use of information

Staff reflected on the potential benefits for patients in terms of how information could be used. For example, patients having the option to access their records might mean that information to support benefit claims and travel insurance applications could be obtained more efficiently. This type of use may have the additional benefit of reducing the staff workload typically associated with these types of requests, but could also have the unintended consequence of increasing patient uncertainty.

Staff generally viewed the opportunity for patients to highlight errors in their EHR favourably, and gave specific examples such as current medication and allergy status. However, it was acknowledged even highlighting these types of errors might not be straightforward due to differences in perceptions between patients and healthcare staff regarding what an error is:

*‘I think when you mention the allergy thing, that’s quite a good one cos in both ways, so sometimes you have things on their, coded, like, where it says they’ve got an allergy but actually it was, they didn’t, it was they were it was vomiting or it wasn’t really an allergy or for some reason it’s not, and it might prevent them having an antibiotic that otherwise could’ve been really useful if they get a chest infection or something else, and equally there might be something missing on there and it might be a severe allergy. So that sort of thing would be really useful if patients could see that and could notify us of any problems there.’ *F2 (Focus group 1)

Much discussion centred on how access to EHRs might be more beneficial for particular groups of patients in the context of safety implications, such as those with a long-term condition or a complex medical history:

*‘… I think to know whether something’s safe you need to know what is expected. So that you should have you blood pressure done every year, so you’d need a list of, I should have my cholesterol done and my… these bloods done and my blood pressure done every twelve months, or if you were diabetic your foot check and all those kind of things. And if they then checked whether they’d had them or not...’ *GP (Focus group 2)

*‘I can see some benefits for certain patients, you know, having their medical records if they’ve got a complex medical story, urm, and having access to information from the past and then, you know, a lot of the time, you know there’s a thing in there, you know, I’ve had this condition for twenty years and you’ve been to a one hour lecture on it, who knows my condition more, the patient will say that...’ *GP Principal (Focus group 4)

#### Historical information

The prospect of historical information becoming visible to patients a significant amount of time later was a key concern raised by staff, for example, there were specific concerns about the use of out-dated terminology. When staff considered these issues, it was clear there was the potential for this to cause anxiety not only for staff, but for patients too.

*‘...I think I worry about them being able to see what has been written in the past when we didn’t know when they would be able to access there, erm because it is easier to be a little bit more mindful about how you might write something, you might still write the same information but you might write it just in case they saw it in a way that was more tactful or something like that because you didn’t have that opportunity in the past, erm so that is of concern...’ *GP (Focus group 3)

*‘...I’m personally fearful, I think it’s a really good idea giving patient’s access to their information, you know, it’s not our information it’s theirs and it relates to them.... I’m very fearful about the consequences on workload with people going through records and coming in complaining about some entry that we made years ago or recent entries that there’s a misinterpretation.....So the actual factual information of a medical record, this is somebody’s story, these are the events that have taken place in their lifetime, this is their bloodgroup, this is their allergies, all that sort of stuff I can see the benefit of. The actual bit about, the, the sort of handwritten notes in effect, just not so sure necessarily how that, urr, can only cause problems really, personally....’ *GP Principal (Focus group 4)

### Theme: changing behaviours and protecting relationships

Staff described how increased patient EHR access could result in changes to staff and patient behaviours.

#### Prompts, reflection and monitoring symptoms

Even subtle changes to staff behaviours in their approach to recording information was said to have the potential to create safety issues. For example, staff described how they often log their general reflections of a consultation to have a record of their thought processes, which may have negative implications if this behaviour of logging reflections were to become restricted. In addition, these notes of thought processes were said to be an important means of communication between staff, often providing additional contextual information and observations, necessary for appropriate actions to be taken. This was viewed as particularly crucial for effective monitoring of symptoms:

*‘It would more, it would cause more problem, if patient can see the, the everything, like the consultations, because I see it as consultations are quite often our thought processes, like this person has a cough for two weeks, if, but has also this as well, they come back I’ll refer the person for, if I put that on then that, and they read it …that automatically that pushes the anxiety of, cos like it’s my thought process but I need to put it on there so that if it’s not me who is seeing the patient next week or the week after they can pick it up and then they do something about it and so it’s just a thought process, but that can lead to significant distress'.’ *GP Partner (Focus group 1)

*‘I guess it is a bit frightening really, I feel, them reading everything that you document and whether they are going to question what you are writing, you have to be really careful what you write’. *Advanced Nurse practitioner (Focus group 3)

#### Professional reputation

Information being recorded in patient notes as a means of protecting professional reputation was also touched upon:

*‘… Yes difficult consultation, long consultation you know and we need to be able to write things that might protect us if things went wrong and there was a complaint or a negligence complaint or something like that and you know so it is important that we write down that we weren’t able to engage with the patient because of this reason very well and you know we tried our best and things like that, which we don’t write in those terms but we will write things like it was a difficult consultation because they had lots of agendas that they wanted to discuss and we wanted to discuss these and things, and then if they read that sort of thing they would probably have a very different perspective on it and would not like to see that we have written those kind of things but they are important for us to write down for our own protection really erm'. *GP (Focus group 3)

#### Unintended consequences – changing patient behaviour

Furthermore, staff worried that patients may limit the information they provide in a consultation through fear of it being on their record in case other people, such as family members, are able to access this. Again, this limiting of sharing information by patients was described as having potentially serious safety implications:

*‘.....then you don’t really know, do you? No one’s ever going to feel safe of coming to the GP practice and saying actually what is the problem or what’s wrong if they’re going away from family members to come here to, like, disclose something, it’s not going to really stay between, within the room is it?’ *Administrator (Dyadic interview)

*‘.....people can ask you why you’ve put something. In fact, somebody today I was just saying to her no one is going to read these records except you and me because she didn’t want me to put that she was stressed....... but people have this paranoia that the big world out there is going to…’ *GP (Focus group 2)

#### Rapport, relationships and sensitivities

Of huge concern to staff was the potential for expanded patient access to EHRs to negatively impact their relationships with patients. Staff described how it was plausible that relationships might be affected due to the communication/recording of information changing. For instance, expanded patient access might lead staff to be less specific in their notes and reflections which they regularly rely upon to ensure a good rapport in consultations:

*‘.....Be less specific, but I don’t think that is helpful because it acts as a really good aide memoire to me that builds my relationship with the patient because I can start the conversation by going “how is your daughter”? “how are they getting on with such and such”?’ *GP (Focus group 3)

Staff generally viewed increased EHR access favourably, for instance – patients being more informed about medications and conditions, however, staff were also concerned that increased access might give rise to friction in the patient - health professional relationship, particularly when patients disagree with information in their record:

*‘Yes in terms of the way you have interpreted something that we have written or as you say some things that they perceive as not being recorded accurately or not, but equally erm it may allow them to be on the same page as us and more trusting so I think there pros and cons of that as well.’ *GP Partner (Focus group 3)

Specific examples were provided where misinterpretation or disagreement from the patient point of view could be detrimental for rapport and patient - health professional relationships, such as allergy coding, and coding relating to alcohol and mental health conditions:

- ***‘Participant 4:*** *Or the coding that we use for alcohol. So we have all sorts of codes for that, problem drinker, alcohol dependent you know*
- ***Participant 3:*** *Alcohol abuse*
- ***Participant 4:*** *Alcohol misuse, all sorts of things and I can imagine that that could be a whole can of worms and could become quite awkward so…’*

Participant 4 - GP Partner; Participant 3 - Advanced Nurse Practitioner (Focus group 3).

In terms of providing feedback via online practice portals, staff felt patients should have the option of providing this anonymously if they prefer. Even so, staff recognised that by providing feedback about care experience via such online portals, even though anonymous, patients may still worry this can be linked back to them in some way and therefore be reluctant to provide feedback:

*‘Yeah and I guess some people, people want to sometimes be anonymous in their feedback and that way it would be, it’s clear who’s given that feedback so it might mean that they don’t openly give feedback through those things.’ *F2 (Focus group 1)

### Theme: secure access and safeguarding

There was extensive discussion and concern surrounding secure access and safeguarding, in particular – how patient confidentiality could be ensured. Much of the concern stemmed from problems that may arise should access not be secure.

#### Secure access and verification

Staff commented that although robust systems could be in place for the initial set-up of patient access to their EHR, such as identity checking and other verification/authentication processes (e.g. NHS Login), it is not always guaranteed who will have access subsequently:

*‘Yeah, but it’s like if we’ve done the online thing here, you’ve given them the records but we don’t know who’s actually going to access those records. There’s no, you know, who have they given that password to and who they’ve given that online access to, because it could be anybody couldn’t it?’ *Practice Nurse (Focus group 2)

Staff also noted the need for a secure verification process to be in place when patients update information online or highlight EHR errors:

*‘.... So if they’ve changed address or, urm, changed their contact number or something. With address we do prefer to have some form of proof, so we, ideally we like them to bring in some kind of proof, but sometimes like if it’s a young mum or something they might have seen the health visitor which they might have seen the proof of update in which case then we’d accept it, cos you know health professional has, has seen the proof, so we’d accept it...’ *Office Manager (Single interview)

#### Safeguarding

Staff identified patients who might be more vulnerable in terms of safeguarding and EHR access, such as in cases of domestic abuse, family separation where children are involved, patients with a mental health condition and elderly patients. The possibility of coercion and/or control was frequently cited within the discussions:

*‘.... but I think there’s an element of… not… coercion’s the wrong word isn’t it? But there’s sort of an element of control and stuff within families and stuff that that’s what you do and… you know, and older people as well. So, you know and that’s fine if that’s, that’s what they want to do and often a lot of us look after our older relatives and older stuff and do stuff, but that step, next step is that you want to go delving around in their notes… urm… I think that you just have to be careful about people who are vulnerable, yeah, and people who can’t read and write…' *GP (Focus group 2)

#### Equitable access

Equitable access was also discussed. It was recognised that many patients rely on family members when accessing online practice services, and this reliance could compound issues of coercion and control. For example, some patients may not have the digital skills to enable then to engage with digital devices:

*‘...they’re not literate with the computers, so they’re dependent on other household members, which means that, you know, they’re going to wait for their son, their grandson, granddaughter, it could be anybody, any member of the family, but it depends on whether they’re going to give them that time to be able to access…’ *Practice Nurse (Focus group 2)

Further examples were provided, including patients whose first language is not English, and patients who may not be able to read and write:

*‘.... And also, it’s sharing your records then with somebody else, I mean, often they come in with somebody else here, but we have interpreters here all the time. So actually, anybody could come in by themselves and not have to bring a family member. Whereas, I suppose if they don’t read and write…’ *GP (Focus group 2)

*‘...of what they’ve found and it could be, I think it could be quite dangerous in that way, because there might have been something that’s mentioned within, within her notes, um, and it could have a detrimental effect on the patient whose notes they are that’s the access is actually… well, because she doesn’t know what’s been, cos if, if she can’t read and write, um, and then it’s husband, it could be a father in law, son, it could be anybody, that’s actually, and so it’s a third member of the family that’s actually looking into the notes.’ *Lead Practice Nurse (Focus group 2)

## Discussion

### Summary

This study explored primary care staff perspectives of patients accessing, viewing and highlighting errors in their EHR and providing feedback via online patient portals. Overall, primary care staff were positive about the prospect of increased patient access and supported the view that patients should have access to their EHR from an ethical standpoint. Several potential benefits were cited as a result of information being more readily accessible to patients, such as the workloads typically associated with requests for information being reduced. However, uncertainties surrounding the parameters of access and information available and what this might look like in the future were discussed, with concerns clustered around the level of information, the use of information and historical information.

Staff reflected on how increased patient EHR access could result in changes to staff and patient behaviours, and that even subtle changes to behaviours had the potential to create safety issues. The potential negative impact on clinician-patient relationships, and the potential mechanisms of this were considered at length, for example, clinicians may be less specific in their notes and reflections which are often relied upon to support rapport in consultations. Of significant concern were secure access, safeguarding, and equitable access. A key reflection was that although secure systems could be in place for the initial set-up of patient access to their EHR, it is not always guaranteed who will have access subsequently, and this was discussed in the context of patients who may be more vulnerable to experiencing coercion and/or controlling behaviour. These findings have the potential to inform the implementation and wider roll-out of patient access to EHRs moving forward.

The findings reinforce previous research of the potential benefits, challenges and negative consequences of enabling patients to access their EHR, resonating particularly with research exploring relational issues. For example, one study concluded that from a clinical perspective, enabling patients to access their record online can support safety behaviours such as correcting information, but also lead to unnecessary patient anxiety, issues with security and confidentiality, equality issues and a risk of coercion [37]. Furthermore, a systematic interpretative review examined the effect of providing patients online access to their EHR and linked transactional services on the provision, quality and safety of healthcare [1]. The review highlighted concerns for clinicians, for instance, the potential to cause unnecessary anxiety, privacy, confidentiality and security. Congruent with our findings, previous research has reported on how patient access to EHRs can result in changes to clinicians’ documentation practices. In particular, our findings support previous research by Blease and colleagues’ and the notion of medical records being used as form of ‘cognitive scaffolding’ to support memory and diagnostic reasoning, and how changes to documentation approaches to minimise or prevent anxiety for patients may undermine a key purpose of record keeping [25]. By taking a qualitative approach, our findings map onto and provide further detail to many of the benefits and challenges proposed by previous research, reinforcing the significance of these issues from a UK perspective. It is important to recognise that at present, most practices do not enable patients to assess the free text information inputted by a clinician. In our work much concern stemmed from the potential for the information available to patients to be expanded in the future (e.g. free-text, historical and prospective information) so many of the scenarios participants were reflecting on were largely hypothetical.

Our study was focused on the primary care staff perspective, but importantly, the findings support previous research from the patient perspective which emphasises the opportunities and challenges of patient access to EHRs. Studies exploring this perspective have reported similar issues; for example secure access and confidentiality, implications for health inequalities, and benefits for self-monitoring [34, 38].

In addition to reinforcing and supporting previous research, our findings provide a more in-depth understanding of the tension between the desired outcome of improving equal access, and some of the potential problems improving access might lead to. For example, whilst equitable access might be an espoused goal of those designing such systems, it does not come without costs, and these need to be articulated and managed. The issues of digital literacy featured significantly in our findings, as participants reflected that not all patients have access to the digital devices to access information, nor the skills to enable them to engage with health information online. Indeed, there is evidence to suggest that some people may be less likely to use online patient portals [39], for example, older adults [40], ethnic minorities [41], people of low socioeconomic status [42], and people with reduced health literacy [43]. However, notably, a viewpoint article highlighted that relational benefits of open notes (reading clinical notes via online patient portals) may be particularly pronounced among portal users who are older, less educated, non-white and whose primary language is not English [44], which underlines that these issues are far from clear cut. Our findings also add important novel details to the more common debates about digital exclusion [45], by suggesting that ‘hidden’ issues such as coercive or controlling behaviour could be compounded by providing access to EHRs. It is important that the research and healthcare communities proceed with caution in implementing these systems, and ensure that full evaluations assess where possible, these ‘hidden’ impacts alongside more established concerns.

### Implications for research and/or practice

The NHS Long Term Plan set out to offer digital-first primary care by 2023/2024 with the aim of offering most patients online tools to access primary care services remotely [46]. As a result of the COVID-19 pandemic, primary care practices and models of care are changing rapidly and this has resulted in an uptake of remote and digital services by patients [26]. NHD Digital^4^ recently set out aims to accelerate patient access to their records via the NHS App, including patients being able to read all new entries in their health record and the ability to request historic coded records. With renewed policy focus and more patients using digital healthcare services in the UK, revisiting the nuance of the challenges and opportunities of increased patient EHR access and incorporating this knowledge into any plans to expand access and any plans for wider implementation, is crucial. Qualitative approaches will be incredibly useful to provide a deeper understanding of key issues such as unintended consequences (positive and negative) experienced by patients and primary care staff resulting from patients accessing their EHRs, and our research makes an important contribution in this regard. Specifically, it is essential for further research to focus on equity, to provide a deeper understanding of the nuanced implications. Furthermore, approaches and processes for errors in EHRs being highlighted by patients should be explored, a user needs approach may prove useful here, to map out the needs of key stakeholders and identify congruence and incongruence in needs whilst considering the digital systems these needs sit within. Finally, the renewed aims and policy focus in the UK to accelerate patient access to EHRs, including plans for patients to be able to read all new entries in their health record and request historic coded records, provides a unique real-time research evaluation opportunity.

### Strengths and limitations

Our study included primary care staff purposively sampled based on practice size and the percentage of patients who used online services. We feel the purposive sampling approach is a strength because it allowed for perspectives to be captured from staff across a wide range of practice types. Furthermore, the diversity of the sample, which included both clinical and non-clinical staff meant there was variability in the discussions and a richer insight into how the topic related to the practice as a whole, as opposed to focusing solely on the clinical perspective. For practical reasons, at two of the practices we were unable to carry out a focus group and instead conducted dyadic and single interviews. Therefore it is probable that the implications for the practice as a whole were not explored as comprehensively as discussion between participants from the same practice was not possible in these instances.

## Conclusion

In addition to reinforcing the findings of previous research conducted outside of UK, our findings also provide unique insights. Primary care staff described being invested and supportive of patients accessing their EHRs, and acknowledged the numerous potential benefits for safety. Uncertainty around the parameters of access, the information available and what this might look like in the future, processes for patients highlighting errors in their records, relational issues, security and safeguarding and equitable access, were key areas warranting examination in future research.

## Data Availability

Not applicable.

## Footnotes

1
https://digital.nhs.uk/services/nhs-app/nhs-app-guidance-for-gp-practices/accelerating-patient-access-to-their-record

2 publically available: https://digital.nhs.uk/data-and-information/data-collections-and-data-sets/data-collections/pomi

3 publically available: https://digital.nhs.uk/data-and-information/data-tools-and-services/data-services/general-practice-data-hub/patients-registered-at-a-gp-practice

4
https://digital.nhs.uk/services/nhs-app/nhs-app-guidance-for-gp-practices/accelerating-patient-access-to-their-record
